# Mortality trends involving fatal-arrhythmias and anemia in the United States: A retrospective analysis of 25 years

**DOI:** 10.1097/MD.0000000000047838

**Published:** 2026-02-20

**Authors:** Sarim Hassan Shahab, Saim Jan, Abdul Wasay, Syeda Malika Naqvi, Muhammad Umar, Muhammad Ahmed, Hadia Ghazala Masood, Abubakar Nazir, Fazal Habib

**Affiliations:** a Department of Internal Medicine, Nishtar Medical University, Multan, Pakistan; b Department of Internal Medicine, Central Park Medical College, Lahore, Pakistan; c Department of Internal Medicine, The Jewish Hospital, Cincinnati, OH; d Department of Medicine, Sher-e-Bangla Medical College, Barisal, Bangladesh.

**Keywords:** anemia, arrhythmia, comorbidity, mortality trends

## Abstract

This study aims to examine long-term trends in age-adjusted mortality rate associated with cardiac arrhythmias among anemic adults in the United States from 1999 to 2023 and assess disparities by sex, race/ethnicity, geographic region, and urban–rural residence. For this retrospective observational study, the data was extracted from the Centers for Disease Control and Prevention Wide-ranging Online Data for Epidemiologic Research. International classification of disease (ICD)-10 codes were used to identify cardiac arrhythmia and anemia. We used Joinpoint Regression Program (V.5.4.0) for the analysis of trends and a *P*-value of <.05 was considered statistically significant. The overall age-adjusted mortality rate for cardiac arrhythmia in anemic patients increased from 2.30 in 1999 to 5.03 in 2023, showing a more than 2-fold rise over the 25-year period. Males consistently had a higher mortality than females, rising from 2.64 to 6.11 in men and from 2.09 to 4.28 in women between 1999 and 2023. Non-Hispanic (NH) White adults had the highest mortality, followed by NH Blacks or African American, Hispanics, and NH others respectively throughout 1999 to 2023. Highest mortality rates were observed in Rhode Island, West Virginia, Ohio, and Vermont. Rural areas had higher mortality than urban areas, rising from 2.52 to 5.63 compared with 2.24 to 4.14 in urban areas between 1999 and 2020. The highest mortality rates were in the midwest and south. In conclusion, mortality from cardiac arrhythmias in anemic adults has increased significantly over the past 25 years. There are disparities by sex, race, region, and rural versus urban setting that suggest the need for focused interventions and targeted policy design for vulnerable communities to avoid preventable deaths.

## 1. Introduction

The human heart is a vital body organ located in the chest cavity. It functions to pump blood throughout the body and has a role in maintaining blood pressure. Electrical impulses from the sinoatrial node control its contractions and relaxations (heart rhythm). Normal heart rhythm is defined as 60 to 100 evenly spaced beats per minute (bpm). Any abnormality in the normal rate, regularity, origin, or conduction path of electrical impulses of the heart is called arrhythmia.^[1]^ It can be further categorized as tachycardia/tachyarrhythmia, bradycardia/bradyarrhythmia, and more. Tachycardia refers to >100 bpm, while bradycardia means <60 bpm.^[2]^ Tachycardia types include atrial fibrillation, atrial flutter, supraventricular tachycardia, ventricular fibrillation, and ventricular tachycardia. Bradycardia can occur due to conduction block or sick sinus syndrome.^[1,3]^ Atrial fibrillation is the most common type, affecting millions globally. Arrhythmia can lead to fatal cardiac symptoms (like stroke, cardiac failure, and sudden cardiac death) and is a major cause of morbidity and mortality worldwide.

Anemia is defined as a condition in which an individual’s hemoglobin concentration and/or red blood cell concentration/number is insufficient to meet his physiological needs. Hemoglobin functions to carry oxygen to the tissues. This inability in anemic patients causes symptoms like fatigue, shortness of breath, palpitations, and conjunctival and palmar pallor. Its severity ranges from an asymptomatic condition to a life-threatening disease. Anemia affects normal cognitive development and growth in children and work productivity in adults and is a major cause of morbidity and mortality in the world.^[4]^ A common cause of anemia is iron deficiency, as iron is an integral ingredient in hemoglobin production.^[5]^ Anemia and iron deficiency have been shown to be common risks for patients with atrial fibrillation. Iron deficiency is often seen in people with chronic cardiac conditions, which leads to adverse clinical outcomes.^[6]^ Prior studies have suggested that anemia increases the risk of new-onset atrial fibrillation. Anemia also increases the risk of bleeding, cardiac events, and total mortality rates in patients with atrial fibrillation.^[7]^

While prior studies have analyzed mortality trends individually for arrhythmia and anemia, no study, to the best of our knowledge, provides the mortality trend analysis of both of these comorbidities together. The mortality trends of these inter-connected diseases need to be studied to help inform policymakers on sound public health policy that strengthens the health system. Data retrieved from the Center of Disease Control (CDC) database allows the analysis of the mortality related to cardiac arrhythmia in anemic adults, with differences based on sex, and ethnicity, and geographical location. It would provide evidence-based health policy and improve resource allocation for vulnerable groups.

## 2. Methods

From 1999 to 2023, data were obtained from the Centers for Disease Control and Prevention’s multiple cause of death files in the Wide-ranging Online Data for Epidemiologic Research (CDC WONDER) database.^[8]^ CDC WONDER is an online communication and information system that operates in the area of public health and provides statistical study data published by the CDC. Deaths related to cardiac arrhythmias and anemias were extracted using the multiple causes of death files focused on adults aged ≥25 years. Individuals younger than 25 years were excluded due to low death counts yielding unstable and unreliable estimates, consistent with CDC WONDER recommendations. The international classification of diseases (ICD)-10 codes for cardiac arrhythmias, I48-49 were used. Anemias was defined using code D50-53 (nutritional anemias), D55-59 (hemolytic anemias), and D60-64 (aplastic anemias). Other literature published in the past used the same ICD-10 codes.^[9-12]^

The research utilized only de-identified and publicly accessible data and thus did not require review by the Institutional Review Board. The observational study was documented in accordance with the strengthening the reporting of observational studies in epidemiology guidelines.^[13]^

### 2.1. Data extraction

Data were obtained for the whole adult population, disaggregated in relation to sex, race/ethnicity, 10-year age bracket (25–34 through ≥85), and US urbanization (metropolitan/nonmetropolitan), the state the person lived in, as well as where the person died. Race and ethnicity started to be documented based on the information reported by the funeral directors and by the next of kin. The race/ethnic composition was categorized into Hispanic (Latino) and non-Hispanic (NH), where the latter was divided into White, Blacks, American Indians, and Asians and Pacific Islanders. According to the United States Census Bureau, the United States had 4 regions, namely northeast, midwest, south, and west.^[14]^ The level of urbanization was operationalized with the help of the urban–rural classification scheme of the National Center of Health Statistics, 2013.^[15]^ Counties that had a population of 50,000 or higher were metropolitan; the others were nonmetropolitan.

We did not use annual data cells with <10 deaths (as recommended by CDC WONDER) because of unstable estimates in the data.^[16]^ This suppression limited the analysis of long-term trends in some subgroups, especially younger age groups and populations of smaller racial/ethnic groups. However, the repressed data is included in the totals collected by CDC WONDER. Thus, they were not left out in the overall analysis. To avoid any technical or human error, 2 authors extracted the data independently. The data was further verified by another author.

### 2.2. Statistical analysis

The CDC WONDER provided annual mortality rates and population data. The direct method of calculating age-adjusted mortality rates (AAMR per 1,00,000 was applied, and AAMRs were standardized to the 2000 US standard population. The AAMRs per 1,00,000 values were provided by the CDC WONDER data base.^[17]^ AAMRs were used to measure race/ethnicity, gender, census region, state, age group and urban–rural classification mortality trends.

Temporal trends were also evaluated with the help of the joinpoint regression relying on the log-linear models that yielded estimates of the annual percent change (APC) values in individual segments and the average annual percent change (AAPC) value of the entire study period. The analyses were carried out with the National Cancer Institute’s Joinpoint Regression software (version 5.4.0).^[18]^ The model started with 1 linear segment, and the addition of joinpoints was done in a statistically significant manner by the Monte Carlo permutation test.^[19]^ For calculation of APC/AAPCs or confidence intervals (CI), parametric method was selected in model selection in joinpoint regression. Joinpoint regression software was used because it is well-suited for detecting temporal trend changes, identifying statistically significant inflection points, and estimating APC and AAPC in long-term mortality data.

The empirically chosen best model fit determined the number and location of joinpoints. A *P*-value < .05 was considered significant. Separate subgroup-specific models were specified based on sex, race/ethnicity, age group, region of the country, urbanization level, and the state of residence. Each segment was reported with APCs and respective 95% CIs.

## 3. Results

During the entire study period, a total of 1,85,935 deaths were reported involving cardiac arrhythmia in anemic patients among US adults. The majority of deaths occurred in medical facilities as inpatients, accounting for 43.3% (n = 80,556) of all deaths. A considerable proportion of deaths occurred at the decedent’s home, representing 21.5% (n = 39,929), while nursing homes or long-term care facilities contributed 27.8% (n = 51,745) of total deaths. Hospice facilities accounted for 3.87% (n = 7202), whereas other locations made up 3.4% (n = 6229). The place of death was unknown for a minimal fraction of cases (0.15%, n = 274; Table S1, Supplemental Digital Content, https://links.lww.com/MD/R445).

### 3.1. Annual trends

A rising overall mortality trend was observed across the study period. The AAMR increased steadily from 2.30 per 1,00,000 in 1999 to 3.51 in 2018 (APC = 1.86; 95% CI: 1.56–2.15), followed by a sharp escalation to 4.99 in 2021 (APC = 13.15; 95% CI: 5.59–21.25), and then plateauing at 5.03 in 2023 (APC = 0.84; 95% CI: −4.55 to 6.54).

**Figure 1.**
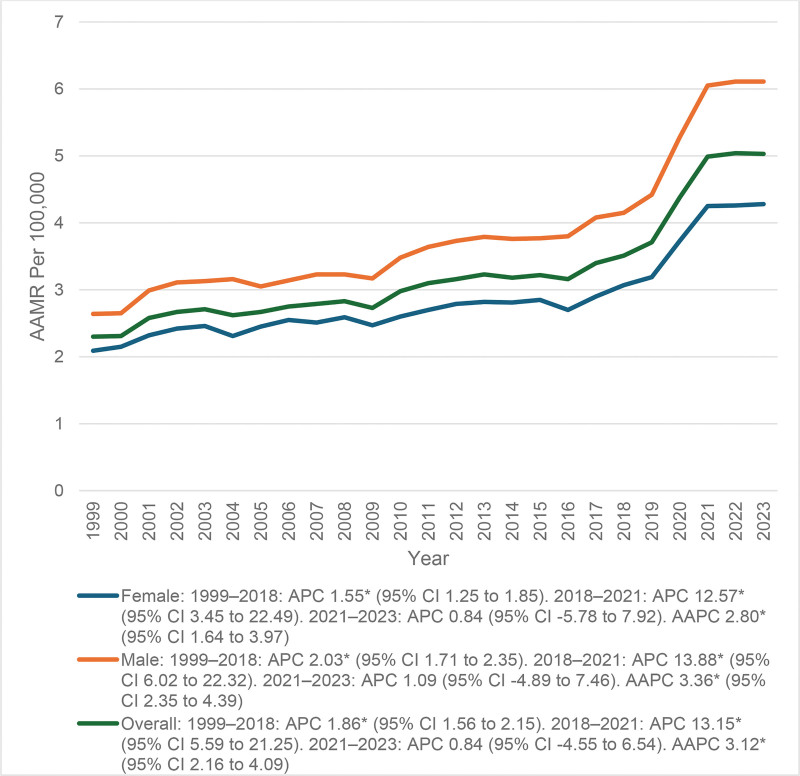
Gender disparities in mortality trends. The “*” sign represents that the trend was statistically significant. AAMR = age-adjusted mortality rate, AAPC = average annual percent change, APC = annual percent change, CI = confidence interval.

The overall long-term trend from 1999 to 2023 remained statistically significant, with an AAPC of 3.12* (95% CI: 2.16–4.09)*; Figure 1 and Table S2, Supplemental Digital Content, https://links.lww.com/MD/R445.

### 3.2. AAMR stratified by sex

Females consistently exhibited lower mortality than males throughout the study period, yet demonstrated a significant long-term increase. The AAMR rose from 2.09 per 1,00,000 in 1999 to 3.07 in 2018 (APC = 1.55*; 95% CI: 1.25–1.85), then sharply increased to 4.25 in 2021 (APC = 12.57*; 95% CI: 3.45–22.49), and remained nearly stable through 2023 at 4.28 (APC = 0.84; 95% CI: −5.78 to 7.92).

The overall long-term trend was statistically significant, with an AAPC of 2.80* (95% CI: 1.64–3.97)*.

Males had consistently higher mortality rates than females, with the AAMR increasing from 2.64 per 1,00,000 in 1999 to 4.15 in 2018 (APC = 2.03*; 95% CI: 1.71–2.35), peaking at 6.05 in 2021 (APC = 13.88*; 95% CI: 6.02–22.32), and stabilizing at 6.11 by 2023 (APC = 1.09; 95% CI: −4.89 to 7.46).

The long-term increase was statistically significant, with an AAPC of 3.36* (95% CI: 2.35–4.39; Fig. 1 and Table S2, Supplemental Digital Content, https://links.lww.com/MD/R445).

### 3.3. AAMR stratified by race

The mortality trend for Hispanic or Latino persons depicted a slowly increasing trend, as demonstrated by the age-adjusted mortality rate (AAMR) increasing from 1.35 per 1,00,000 in 1999 to 2.10 in 2018 (APC = 1.71*; 95% CI: 0.73–2.69). The AAMR increased sharply, yet not statistically significant, between 2018 and 2021 in which there was an AAMR of 3.29 in 2021 (APC = 14.11; 95% CI: –5.64 to 37.99) followed by a slight decrease to an AAMR of 3.03 in 2023 (APC = –4.34; 95% CI: –19.45 to 13.60). The long-term trend showed a marginally significant increase in the AAMR for Hispanic or Latino persons, (AAPC of 2.65; 95% CI: –0.05 to 5.44) which suggests there was a gradual increase despite fluctuations in short-term trends.

Initially, NH Black or African American individuals demonstrated a trend that was stable to slightly decreasing where AAMR fluctuated between approximately 3.0 per 1,00,000 to 2.91 per 1,00,000 between 1999 and 2017 (APC = –0.33; 95% CI: −1.05 to 0.40). From 2017, however, there was a significant upward trend, a statistically significant increase driving the average annual percentage change (AAPC) to 4.99 by 2023 (APC = 10.46*; 95% CI: 7.04–13.94). There was an overall significant increase when looking longer-term with an average annual percentage change (AAPC) of 2.27* (95% CI: 1.42–3.12).

NH White individuals consistently exhibited the highest mortality burden across all racial groups. The AAMR increased steadily from 2.21 in 1999 to 3.64 in 2018 (APC = 2.21*; 95% CI: 1.91–2.52), followed by a sharp rise to 5.21 in 2021 (APC = 13.18*; 95% CI: 5.77–21.11), and then stabilized at 5.22 in 2023 (APC = 0.96; 95% CI: –5.58 to 7.97). The overall trend from 1999 to 2023 showed a significant long-term increase, with an AAPC of 3.42* (95% CI: 2.42–4.42).

**Figure 2.**
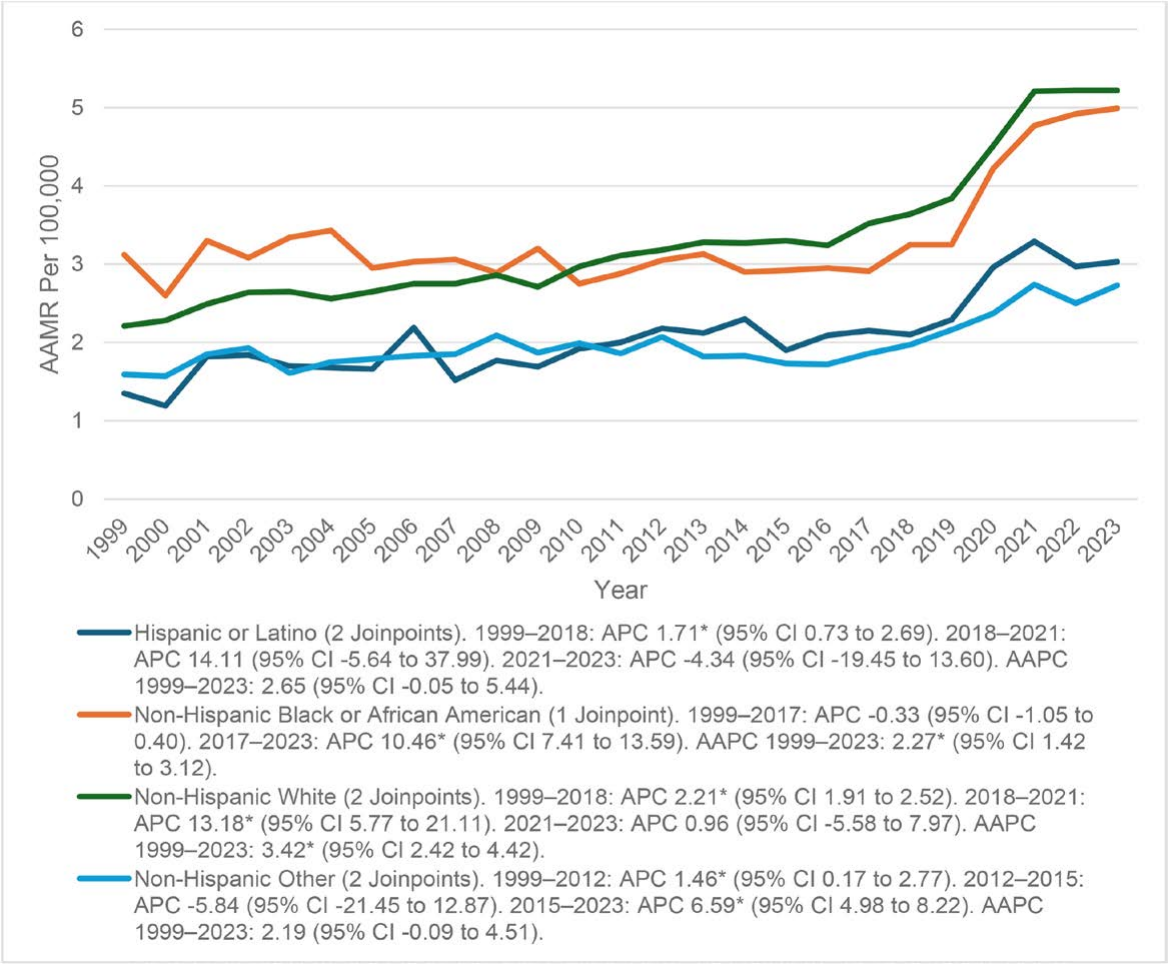
Racial disparities in mortality trends. The “*” sign represents that the trend was statistically significant. AAMR = age-adjusted mortality rate, AAPC = average annual percent change, APC = annual percent change, CI = confidence interval.

NH other racial groups experienced fluctuating but upward trends. The AAMR increased from 1.59 in 1999 to 2.07 in 2012 (APC = 1.46*; 95% CI: 0.17–2.77), briefly declined from 2012 to 2015 (APC = –5.84; 95% CI: –21.45 to 12.87), and then rose significantly from 2015 to 2023, reaching 2.73 in 2023 (APC = 6.59*; 95% CI: 4.98–8.22). The long-term trend showed a borderline significant increase, with an AAPC of 2.19 (95% CI: –0.09 to 4.51; Fig. 2 and Table S3, Supplemental Digital Content, https://links.lww.com/MD/R445).

### 3.4. AAMR stratified by census region

**Figure 3.**
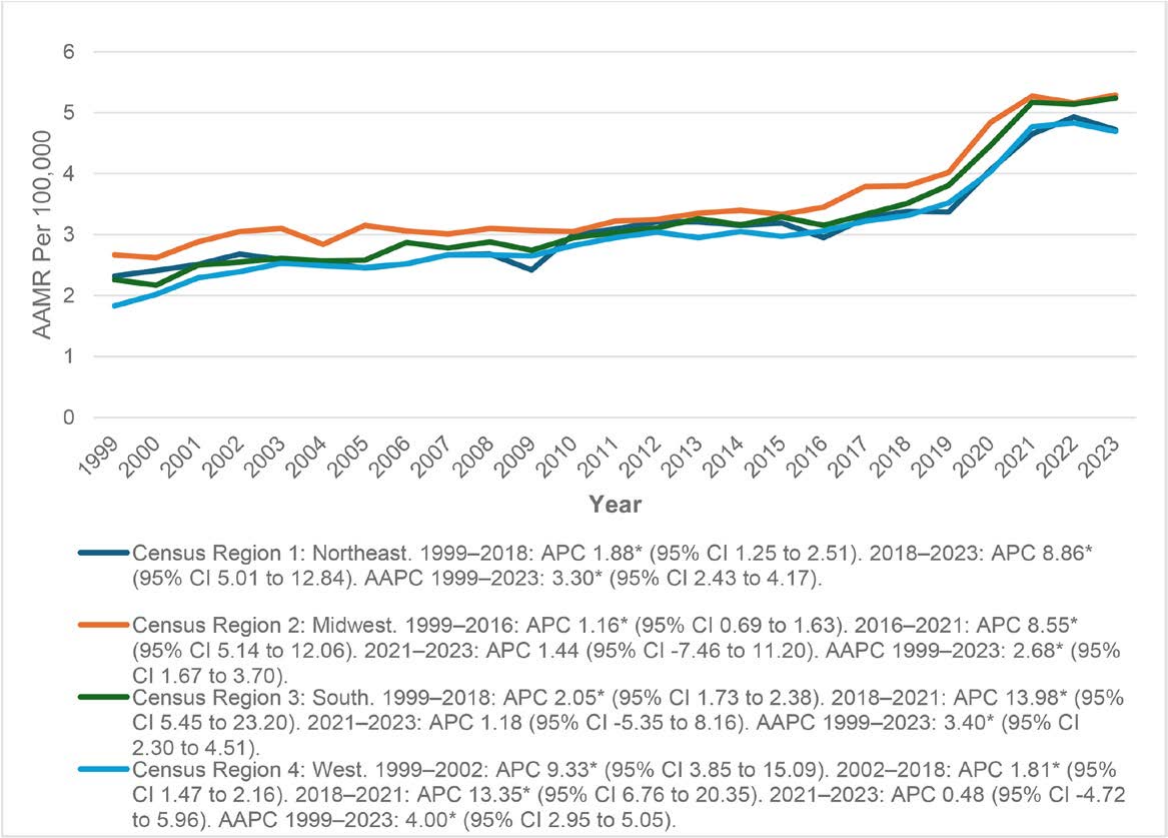
Census region stratified mortality trends. The “*” sign represents that the trend was statistically significant. AAMR = age-adjusted mortality rate, AAPC = average annual percent change, APC = annual percent change, CI = confidence interval.

In all US census regions AAMR exhibited a similar pattern with an increase around 2020 to 2021 and slower growth after 2021. The Northeast had a sustained increase in AAMR over the long-term with a large increase after 2018 (an AAPC = 3.30*; 95% CI: 2.43–4.17). The midwest showed moderate increases in AAMR until 2016, experienced a rapid increase from 2016 to 2021 and relative stability afterwards (an AAPC = 2.68*; 95% CI: 1.67–3.70). The south was characterized by persistently high AAMR with consistent increases until 2018, a large increase from 2018 to 2021, and slower growth after 2021 (an AAPC = 3.40*; 95% CI: 2.30–4.51). The west showed the steepest rise overall, early sharp increase, and consistent long-term growth with a gradual increase after 2018, to plateau from 2021 to 2023, with an AAPC = 4.00* (95% CI: 2.95–5.05), the highest of any region. In general, all regions demonstrated long-term increases in AAMR over the time periods assessed with statistically significant increases overall (Fig. 3 and Table S4, Supplemental Digital Content, https://links.lww.com/MD/R445).

### 3.5. AAMR stratified by urbanization

Urban (metropolitan) areas demonstrated a gradual rise in mortality due to cardiac arrhythmia in anemic patients with the AAMR increasing from 2.24 per 1,00,000 in 1999 to 3.35 in 2018 (APC 1.7796* [95% CI: 1.4679–2.0923]). This was followed by a sharp increase to 4.14 in 2020 (APC 11.4974* [95% CI: 3.5185–20.0914]). The overall long-term trend was statistically significant, with an AAPC of 2.6674* (95% CI: 1.9454–3.3945).

**Figure 4.**
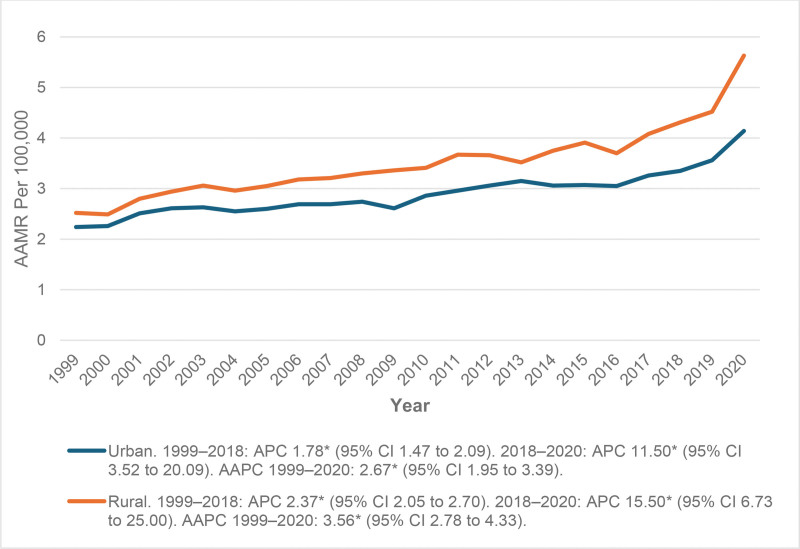
Disparities in mortality trends between urban and rural areas (1999–2020). The “*” sign represents that the trend was statistically significant. AAMR = age-adjusted mortality rate, AAPC = average annual percent change, APC = annual percent change, CI = confidence interval.

Rural (nonmetropolitan) areas consistently exhibited higher mortality rates throughout the study period, rising from 2.52 in 1999 to 4.31 in 2018 APC 2.37* (95% CI: 2.04–2.70), followed by a notable surge to 5.63 in 2020 with an APC 15.50* (95% CI: 6.73–25.00). The long-term trend was also statistically significant, with an AAPC of 3.55* (95% CI: 2.78–4.33; Fig. 4 and Table S5, Supplemental Digital Content, https://links.lww.com/MD/R445).

### 3.6. States stratified trends

**Figure 5.**
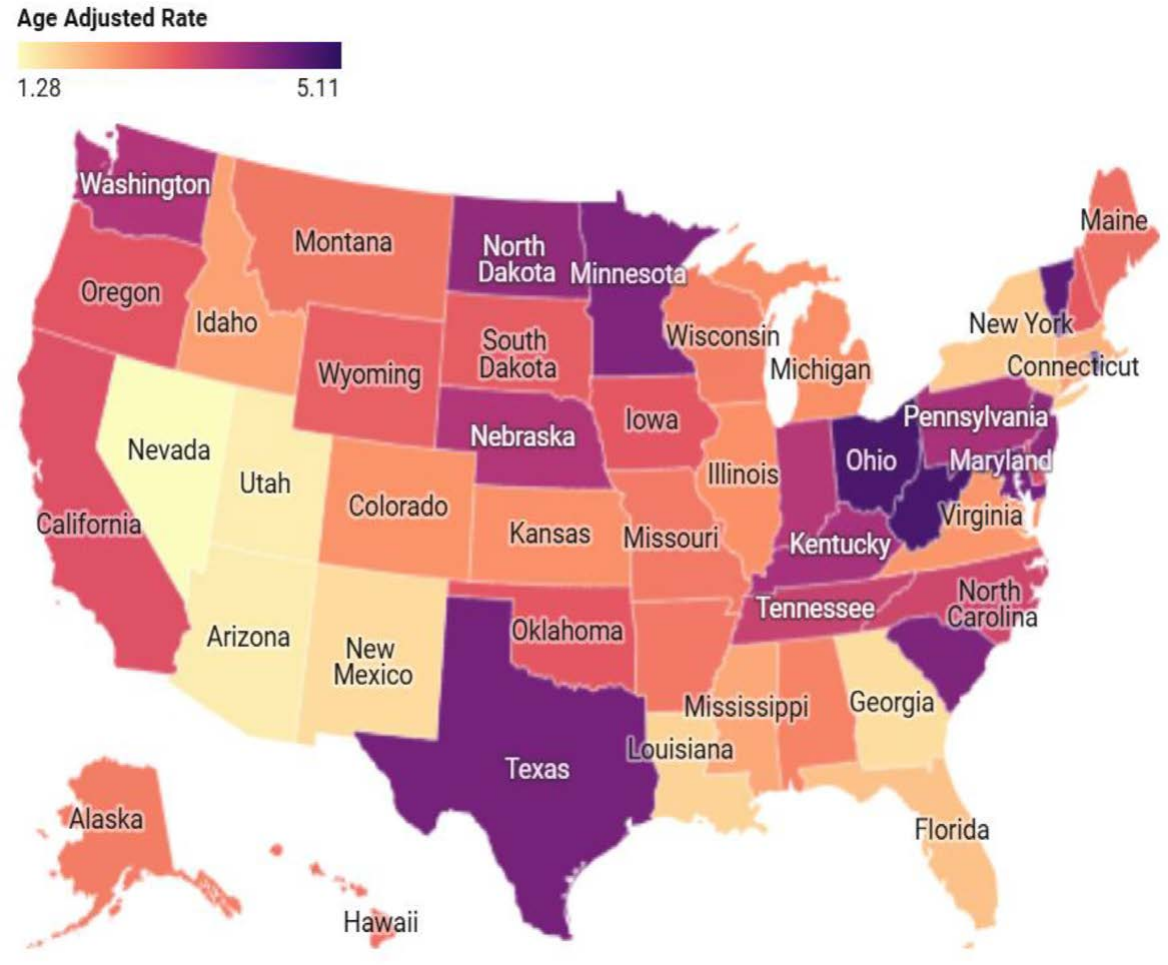
State specified mortality trends involving cardiac arrhythmia in anemic adults (1999–2020).

Across the United States, notable variation in AAMRs due to cardiac arrhythmia in anemic patients was observed among states. The highest AAMR was reported in Rhode Island (5.11 per 1,00,000 population), followed by West Virginia (4.82), Ohio (4.80), Texas (4.37), and Maryland (4.39). Other states with elevated rates included Minnesota (4.28), South Carolina (4.28), and Vermont (4.61). In contrast, the lowest mortality rates were observed in Nevada (1.28), Utah (1.46), Arizona (1.47), New Mexico (1.67), and Georgia (1.65; Fig. 5 and Table S6, Supplemental Digital Content, https://links.lww.com/MD/R445).

## 4. Discussion

During the study, 1,85,935 deaths from cardiac arrhythmia occurred in anemic adults in the United States. The place of death data showed that the majority (43.3%) of the deaths occurred in a medical institution as inpatients, meaning that most of the patients died under an active clinical setting. A substantial portion of deaths (21.5%) occurred at home, implying that most patients either experienced fatal arrhythmia at home, and outside of an organized care option, or did not have direct access to arranged medical treatment. This data suggests the ongoing challenges when detecting, monitoring, and initiating treatment in the out of hospital setting. Moreover, the fact that 27.8% of deaths occur in a nursing home or long-term care facility indicates the extra vulnerability of older patients or those with chronic disease with anemia that are at high risk for cardiac complications.

The data shows that anemic cardiac patients face challenges in both hospital and community settings, and that arrhythmia-related deaths also occur outside hospitals. Advancement in cardiovascular care have made earlier improvements in mortality but subsequent rising mortality reflects rising comorbidity burden and shifting healthcare practices.^[20]^

The overall AAMR increased from 2.30 in 1999 to 5.03 in 2023. This rising trend is likely due to the aging population and advancements in arrhythmia diagnoses. Mortality increased from 4.99 per 1,00,000 populations from 2018 to 2021, which aligns with the COVID-19 pandemic era, where patients were more vulnerable and had poor outcomes. The pandemic-caused physiological and health disturbances created a sudden spike in mortality in 2021, then returned to an increase that was more gradual. Mortality flattened after this peak in 2023, with evidence of stabilization as healthcare systems shifted and control of chronic disease returned.^[21]^ Due to the results demonstrating a sustained increase in arrhythmia-related mortality in anemic patients, it is suggested that screening for anemia should be included as part of cardiac risk assessment.^[20]^ Gender differences were also significant over the course of the study. Both sexes displayed a clear upward trend in mortality over time, although females consistently had lower mortality rates than males.^[22]^ The AAMR for women rose slowly over time, from 1999 up to 2018, indicating a gradual but steady increase in the disease burden. While women began at a lower baseline level of mortality, they had a steady increase over the course of the study. By contrast, mortality was consistently higher for men as compared to women. This correlation is indicative of a closer link between male cardiovascular susceptibility, anemia, and arrhythmogenic risk.^[23]^ The resultant inequality can be explained in part by biological processes, such as the effects of testosterone on cardiac electrophysiology and increased comorbidities that pose a higher risk of mortality. Sex-based inequalities in healthcare seeking and delayed treatment of anemia can also be the reason behind these ongoing sex-based inequalities in arrhythmia-related deaths.^[24,25]^ Therefore, sex-specific preventive strategies that focus on arrhythmia control, anemia correction, and equitable long-term cardiac management are essential to improve overall outcomes. In the US, there were significant racial and ethnic disparities in the mortality rate of cardiac arrhythmia among anemic adults. AAMRs rose consistently from 1999 to 2023, with the highest mortality burden being among NH White individuals across the study period.^[26]^ NH Black or African American accounted for somewhat steady rates initially but, after 2017, their mortality rate rose sharply in 2023.

Despite their rising mortality rates over time from 1999 to 2023, Hispanic or Latino individuals reported the lowest all-cause mortality rates. Lower smoking prevalence, stronger social and family connections, and improved eating or lifestyle habits are all potential protective factors.^[27]^

The NH portion of “Other” racial category had a generally rising but unstable trend, which indicates that populations of smaller sizes are becoming vulnerable to mortality due to arrhythmia.

In terms of geographic location, the all the regions displayed a gradual rising trend. It is likely that this corresponds to regional variation in access to specialized care, assessment of anemia, and management of cardiovascular disease. The higher mortality of the south may be attributable to a combination of socioeconomic inequities, increased comorbid burden, and limited access to care.^[28]^ Mortality steadily increased until 2016, but from 2016 to 2021, there was a rapid increase possibly due to delays in treatment or effects of the healthcare disruptions from the pandemic.^[29]^ The ongoing regional increase suggests congruently that arrhythmia mortality burden associated with anemia among adults is a national public health concern in need of focused interventions, and not limited to specific geographic areas. While the subsequent stabilization is indicative of a recovery considering service delivery, the overall increase in 2020 to 2021, at least in part, is likely observational of systemic healthcare impacts of COVID-19. All of these results show that mortality continues to gradually increase nationwide, thus development and implementation of stronger and region-based anemia/cardiac care policies will be required.

Significant long-term increases occurred in both regions but more so in rural ones. In urban areas, the AAMR rose from 2.24 in 1999 to 3.35 in 2018 and then sharply rose to 4.14 in 2020. Similar mortality pattern was also observed in rural areas, but rural areas had a consistently higher mortality rate than urban areas, increased awareness of diagnostic measures, an aging population, and disruptions in health care due to the COVID-19 pandemic could all have contributed to the observed patterns.^[30]^ The notable difference in trends could be due to multiple factors including poor access to specialty cardiac and hematologic services, insufficient health care resources, increased comorbidity burden, and financial challenges in rural regions. These patterns highlight the immediate need for better access to rural health care facilities, the development of early screening initiatives, and the enhancement of equitable access to treatment for cardiac disease and anemia in an effort to narrow the equitable gap in mortality trends between urban and rural populations.^[31]^

Rhode Island, West Virginia, and Ohio had the highest rates of mortality from cardiac arrhythmias. Decreased mortality rates in Arizona and Utah suggest that there are increased avenues of preventive care and increased healthy habits not as easily found in other areas. Higher AAMR in West Virginia and Ohio may suggest increased risk factors for cardiovascular disease. These data indicate there is a need for disease specific interventions and targeted prevention strategies for better management of cardiac arrhythmias in anemic. Lastly, it is indicated that disparities in mortality outcomes are still driven by systemic inequity and the resulting unequal access to preventive cardiac care. Interventions that target and improve access to early diagnoses, effectively treat comorbidity and access to cardiac care will be needed to address inequitable mortality disparity.

## 5. Limitations

There are a number of limitations in this study. First, the use of death certificate data on CDC WONDER database will provide a chance of misclassification or underreporting of arrhythmias (ICD-10 codes I48-49) and anemias (ICD-10 code D50-53, D55-59, and D60-64) as underlying or contributing causes of death. Second, the database is not that detailed clinically, such as disease stage, history of treatment, comorbidities, and socioeconomic determinants of health, limiting the level of interpretation. Third, reporting of race and ethnicity on death certificates could be misclassified especially among American Indian/Alaska Native and Asian or Pacific Islander, thus biasing the subgroup analysis. Fourth, the inconsistency in the coding and classification could have been brought by the variation in death certificate reporting practices across states and times. Fifth, the COVID-19 pandemic could have indirectly impacted care continuity and healthcare seeking behavior and could result in influencing mortality trends in 2020 to 2021. Furthermore, we did not classify the type of arrhythmia or the type of anemia to avoid unreliable data sets, had the data been available, the study would have been more rigorous. However, we encourage researchers to analyze the data for each type of arrhythmia and anemia separately in future. Furthermore, since it is an ecological, mortality-based study, no association can be construed as causal. Lastly, the reported mortality trends should be interpreted as population-level indicators rather than precise estimates of individual risk.

## 6. Conclusion and future directions

The mortality rate associated with cardiac arrhythmia among anemic patients increased markedly, more than doubled, from 1999 to 2023, in US adults. Increased mortality burden remained higher among males than females, while NH Black individuals exhibited the highest mortality. The greatest mortality rates were observed in southern and western regions and among rural populations.

This study emphasizes that the anemic individuals should receive regular cardiac monitoring to enable early detection and management of cardiac arrhythmias. Targeted prevention strategies should focus on the individuals of those regions bearing highest mortality burden. Improvement in disease surveillance, equitable access to cardiovascular prevention and treatment to reduce the mortality burden among vulnerable populations is necessary. Public health strategies should focus on improving access to diagnostic and specialty care in rural and high-burden regions. Strengthened surveillance and standardized mortality reporting may help reduce disparities and prevent avoidable arrhythmia-related deaths.

## Author contributions

**Conceptualization:** Sarim Hassan Shahab, Saim Jan, Muhammad Ahmed.

**Data curation:** Sarim Hassan Shahab, Saim Jan.

**Formal analysis:** Sarim Hassan Shahab, Abdul Wasay.

**Investigation:** Sarim Hassan Shahab, Abdul Wasay.

**Methodology:** Abdul Wasay.

**Project administration:** Sarim Hassan Shahab, Saim Jan, Abubakar Nazir.

**Resources:** Saim Jan.

**Software:** Syeda Malika Naqvi.

**Supervision:** Syeda Malika Naqvi, Abubakar Nazir.

**Validation:** Sarim Hassan Shahab, Abdul Wasay, Syeda Malika Naqvi.

**Visualization:** Sarim Hassan Shahab, Muhammad Umar, Hadia Ghazala Masood.

**Writing – original draft:** Sarim Hassan Shahab, Saim Jan, Abdul Wasay, Syeda Malika Naqvi, Muhammad Umar, Muhammad Ahmed, Hadia Ghazala Masood, Fazal Habib.

**Writing – review & editing:** Sarim Hassan Shahab, Saim Jan, Abdul Wasay, Syeda Malika Naqvi, Muhammad Umar, Muhammad Ahmed, Hadia Ghazala Masood, Abubakar Nazir, Fazal Habib.

## Supplementary Material
