# Supplementary material for: Mortality trends involving fatal-arrhythmias and anemia in the United States: A retrospective analysis of 25 years
Source: Medicine (Baltimore). 2026 Feb 20;105(8):e47838. doi: 10.1097/MD.0000000000047838 (PMC12928970; doi:10.1097/MD.0000000000047838)
Supplement: Supplementary file 1 [file medi-105-e47838-s001.docx]

**Supplementary Table 1**: Place of death data table Involving Fatal-Arrhythmias and Anemia in the United States

| **Place of death** | **Number of death** | **% total** |
| --- | --- | --- |
| Medical Facility | 80,556 | 43.32% |
| Decedent's home | 39929 | 21.43% |
| Hospice facility | 7202 | 3.87% |
| Nursing home/long term care | 51745 | 27.83% |
| Other | 6229 | 3.35% |
| Place of death unknown | 274 | 0.14% |
| TOTAL | **185,935** | 100% |

# **Supplementary Table 2:** Gender stratified and overall mortality data Involving Fatal-Arrhythmias and Anemia in the United States

| **Year** | **Sex** | **Age Adjusted Rate (95% CI)** | **Sex2** | **Age Adjusted Rate (95% CI)3** | **Overall** | **Age Adjusted Rate (95% CI)4** |
| --- | --- | --- | --- | --- | --- | --- |
| 1999 | Female | 2.09 (2.01–2.18) | Male | 2.64 (2.51–2.77) | Overall (M+F) | 2.30 (2.23–2.37) |
| 2000 | Female | 2.15 (2.06–2.24) | Male | 2.65 (2.52–2.78) | Overall (M+F) | 2.31 (2.24–2.38) |
| 2001 | Female | 2.32 (2.23–2.40) | Male | 2.99 (2.86–3.13) | Overall (M+F) | 2.58 (2.50–2.65) |
| 2002 | Female | 2.42 (2.33–2.51) | Male | 3.11 (2.97–3.24) | Overall (M+F) | 2.67 (2.59–2.74) |
| 2003 | Female | 2.46 (2.37–2.55) | Male | 3.13 (2.99–3.27) | Overall (M+F) | 2.71 (2.64–2.79) |
| 2004 | Female | 2.31 (2.22–2.40) | Male | 3.16 (3.03–3.30) | Overall (M+F) | 2.62 (2.54–2.69) |
| 2005 | Female | 2.45 (2.36–2.54) | Male | 3.05 (2.92–3.18) | Overall (M+F) | 2.67 (2.60–2.75) |
| 2006 | Female | 2.55 (2.46–2.64) | Male | 3.14 (3.01–3.27) | Overall (M+F) | 2.75 (2.68–2.83) |
| 2007 | Female | 2.51 (2.42–2.60) | Male | 3.23 (3.10–3.36) | Overall (M+F) | 2.79 (2.72–2.87) |
| 2008 | Female | 2.59 (2.50–2.68) | Male | 3.23 (3.10–3.35) | Overall (M+F) | 2.83 (2.76–2.90) |
| 2009 | Female | 2.47 (2.38–2.56) | Male | 3.17 (3.04–3.29) | Overall (M+F) | 2.73 (2.66–2.80) |
| 2010 | Female | 2.60 (2.52–2.69) | Male | 3.48 (3.35–3.61) | Overall (M+F) | 2.98 (2.90–3.05) |
| 2011 | Female | 2.70 (2.61–2.79) | Male | 3.64 (3.51–3.77) | Overall (M+F) | 3.10 (3.03–3.18) |
| 2012 | Female | 2.79 (2.70–2.88) | Male | 3.73 (3.60–3.86) | Overall (M+F) | 3.16 (3.09–3.24) |
| 2013 | Female | 2.82 (2.73–2.91) | Male | 3.79 (3.66–3.92) | Overall (M+F) | 3.23 (3.15–3.30) |
| 2014 | Female | 2.81 (2.72–2.90) | Male | 3.76 (3.63–3.88) | Overall (M+F) | 3.18 (3.11–3.26) |
| 2015 | Female | 2.85 (2.76–2.94) | Male | 3.77 (3.64–3.89) | Overall (M+F) | 3.22 (3.15–3.29) |
| 2016 | Female | 2.70 (2.62–2.79) | Male | 3.80 (3.68–3.93) | Overall (M+F) | 3.16 (3.08–3.23) |
| 2017 | Female | 2.90 (2.81–2.99) | Male | 4.08 (3.95–4.20) | Overall (M+F) | 3.40 (3.32–3.47) |
| 2018 | Female | 3.07 (2.98–3.16) | Male | 4.15 (4.03–4.28) | Overall (M+F) | 3.51 (3.44–3.59) |
| 2019 | Female | 3.19 (3.10–3.28) | Male | 4.42 (4.29–4.55) | Overall (M+F) | 3.71 (3.64–3.79) |
| 2020 | Female | 3.73 (3.64–3.83) | Male | 5.28 (5.14–5.41) | Overall (M+F) | 4.38 (4.30–4.46) |
| 2021 | Female | 4.25 (4.14–4.36) | Male | 6.05 (5.90–6.20) | Overall (M+F) | 4.99 (4.91–5.08) |
| 2022 | Female | 4.26 (4.15–4.36) | Male | 6.11 (5.96–6.25) | Overall (M+F) | 5.04 (4.96–5.13) |
| 2023 | Female | 4.28 (4.18–4.38) | Male | 6.11 (5.96–6.25) | Overall (M+F) | 5.03 (4.95–5.12) |

**Supplementary Table 3:** Race stratified mortality data Involving Fatal-Arrhythmias and Anemia in the United States

| **Year** | **Race** | **Age Adjusted Rate (95% CI)** | **Year2** | **Race3** | **Age Adjusted Rate (95% CI)4** |
| --- | --- | --- | --- | --- | --- |
| 1999 | NH Black or African American | 3.12 (2.83–3.41) | 1999 | NH White | 2.21 (2.14–2.28) |
| 2000 | NH Black or African American | 2.60 (2.33–2.86) | 2000 | NH White | 2.28 (2.20–2.35) |
| 2001 | NH Black or African American | 3.30 (3.01–3.60) | 2001 | NH White | 2.49 (2.41–2.57) |
| 2002 | NH Black or African American | 3.08 (2.80–3.37) | 2002 | NH White | 2.64 (2.56–2.71) |
| 2003 | NH Black or African American | 3.34 (3.04–3.63) | 2003 | NH White | 2.65 (2.57–2.72) |
| 2004 | NH Black or African American | 3.43 (3.13–3.72) | 2004 | NH White | 2.56 (2.49–2.64) |
| 2005 | NH Black or African American | 2.95 (2.68–3.22) | 2005 | NH White | 2.65 (2.57–2.72) |
| 2006 | NH Black or African American | 3.03 (2.76–3.31) | 2006 | NH White | 2.75 (2.67–2.83) |
| 2007 | NH Black or African American | 3.06 (2.79–3.33) | 2007 | NH White | 2.75 (2.68–2.83) |
| 2008 | NH Black or African American | 2.89 (2.63–3.15) | 2008 | NH White | 2.86 (2.78–2.93) |
| 2009 | NH Black or African American | 3.20 (2.93–3.47) | 2009 | NH White | 2.71 (2.64–2.79) |
| 2010 | NH Black or African American | 2.75 (2.50–2.99) | 2010 | NH White | 2.97 (2.89–3.05) |
| 2011 | NH Black or African American | 2.88 (2.63–3.13) | 2011 | NH White | 3.11 (3.03–3.19) |
| 2012 | NH Black or African American | 3.05 (2.80–3.30) | 2012 | NH White | 3.18 (3.10–3.26) |
| 2013 | NH Black or African American | 3.13 (2.88–3.38) | 2013 | NH White | 3.28 (3.20–3.36) |
| 2014 | NH Black or African American | 2.90 (2.67–3.13) | 2014 | NH White | 3.27 (3.19–3.35) |
| 2015 | NH Black or African American | 2.92 (2.69–3.15) | 2015 | NH White | 3.30 (3.22–3.38) |
| 2016 | NH Black or African American | 2.95 (2.72–3.18) | 2016 | NH White | 3.24 (3.16–3.32) |
| 2017 | NH Black or African American | 2.91 (2.69–3.14) | 2017 | NH White | 3.52 (3.44–3.59) |
| 2018 | NH Black or African American | 3.25 (3.01–3.48) | 2018 | NH White | 3.64 (3.56–3.72) |
| 2019 | NH Black or African American | 3.25 (3.03–3.48) | 2019 | NH White | 3.84 (3.76–3.92) |
| 2020 | NH Black or African American | 4.22 (3.96–4.47) | 2020 | NH White | 4.51 (4.43–4.60) |
| 2021 | NH Black or African American | 4.77 (4.49–5.04) | 2021 | NH White | 5.21 (5.11–5.30) |
| 2022 | NH Black or African American | 4.92 (4.65–5.20) | 2022 | NH White | 5.22 (5.13–5.31) |
| 2023 | NH Black or African American | 4.99 (4.72–5.27) | 2023 | NH White | 5.22 (5.13–5.32) |
| 1999 | NH Other | 1.59 (1.22–2.04) | 1999 | Hispanic or Latino | 1.35 (1.08–1.63) |
| 2000 | NH Other | 1.57 (1.20–2.01) | 2000 | Hispanic or Latino | 1.19 (0.96–1.47) |
| 2001 | NH Other | 1.85 (1.47–2.31) | 2001 | Hispanic or Latino | 1.82 (1.52–2.13) |
| 2002 | NH Other | 1.93 (1.55–2.38) | 2002 | Hispanic or Latino | 1.84 (1.54–2.14) |
| 2003 | NH Other | 1.61 (1.27–2.01) | 2003 | Hispanic or Latino | 1.70 (1.43–1.97) |
| 2004 | NH Other | 1.75 (1.41–2.15) | 2004 | Hispanic or Latino | 1.68 (1.41–1.94) |
| 2005 | NH Other | 1.79 (1.44–2.14) | 2005 | Hispanic or Latino | 1.66 (1.40–1.92) |
| 2006 | NH Other | 1.83 (1.49–2.17) | 2006 | Hispanic or Latino | 2.19 (1.90–2.49) |
| 2007 | NH Other | 1.85 (1.52–2.18) | 2007 | Hispanic or Latino | 1.52 (1.28–1.75) |
| 2008 | NH Other | 2.09 (1.75–2.43) | 2008 | Hispanic or Latino | 1.77 (1.52–2.01) |
| 2009 | NH Other | 1.87 (1.56–2.18) | 2009 | Hispanic or Latino | 1.69 (1.46–1.93) |
| 2010 | NH Other | 1.99 (1.67–2.31) | 2010 | Hispanic or Latino | 1.92 (1.67–2.16) |
| 2011 | NH Other | 1.86 (1.56–2.15) | 2011 | Hispanic or Latino | 2.00 (1.77–2.24) |
| 2012 | NH Other | 2.07 (1.78–2.37) | 2012 | Hispanic or Latino | 2.18 (1.94–2.42) |
| 2013 | NH Other | 1.82 (1.55–2.08) | 2013 | Hispanic or Latino | 2.12 (1.89–2.35) |
| 2014 | NH Other | 1.83 (1.57–2.08) | 2014 | Hispanic or Latino | 2.30 (2.07–2.53) |
| 2015 | NH Other | 1.73 (1.49–1.97) | 2015 | Hispanic or Latino | 1.90 (1.70–2.10) |
| 2016 | NH Other | 1.72 (1.49–1.95) | 2016 | Hispanic or Latino | 2.09 (1.88–2.30) |
| 2017 | NH Other | 1.86 (1.62–2.10) | 2017 | Hispanic or Latino | 2.15 (1.95–2.36) |
| 2018 | NH Other | 1.97 (1.74–2.21) | 2018 | Hispanic or Latino | 2.10 (1.90–2.30) |
| 2019 | NH Other | 2.16 (1.92–2.39) | 2019 | Hispanic or Latino | 2.29 (2.08–2.49) |
| 2020 | NH Other | 2.37 (2.13–2.61) | 2020 | Hispanic or Latino | 2.96 (2.73–3.18) |
| 2021 | NH Other | 2.74 (2.47–3.01) | 2021 | Hispanic or Latino | 3.29 (3.05–3.52) |
| 2022 | NH Other | 2.50 (2.25–2.74) | 2022 | Hispanic or Latino | 2.97 (2.75–3.19) |
| 2023 | NH Other | 2.73 (2.48–2.98) | 2023 | Hispanic or Latino | 3.03 (2.81–3.24) |

**Supplementary Table 4:** Census region specified mortality data Involving Fatal-Arrhythmias and Anemia in the United States

| **Year** | **Census Region** | **Age Adjusted Rate (95% CI)** | **Year** | **Census Region** | **Age Adjusted Rate (95% CI)** |
| --- | --- | --- | --- | --- | --- |
| 1999 | Census Region 1: Northeast | 2.32 (2.16–2.47) | 1999 | Census Region 2: Midwest | 2.67 (2.51–2.82) |
| 2000 | Census Region 1: Northeast | 2.41 (2.26–2.57) | 2000 | Census Region 2: Midwest | 2.62 (2.47–2.78) |
| 2001 | Census Region 1: Northeast | 2.51 (2.35–2.66) | 2001 | Census Region 2: Midwest | 2.88 (2.72–3.04) |
| 2002 | Census Region 1: Northeast | 2.68 (2.52–2.84) | 2002 | Census Region 2: Midwest | 3.05 (2.89–3.21) |
| 2003 | Census Region 1: Northeast | 2.59 (2.43–2.75) | 2003 | Census Region 2: Midwest | 3.10 (2.94–3.26) |
| 2004 | Census Region 1: Northeast | 2.55 (2.39–2.71) | 2004 | Census Region 2: Midwest | 2.84 (2.68–2.99) |
| 2005 | Census Region 1: Northeast | 2.45 (2.30–2.61) | 2005 | Census Region 2: Midwest | 3.15 (2.99–3.32) |
| 2006 | Census Region 1: Northeast | 2.52 (2.36–2.67) | 2006 | Census Region 2: Midwest | 3.06 (2.90–3.22) |
| 2007 | Census Region 1: Northeast | 2.67 (2.51–2.83) | 2007 | Census Region 2: Midwest | 3.01 (2.85–3.16) |
| 2008 | Census Region 1: Northeast | 2.68 (2.52–2.83) | 2008 | Census Region 2: Midwest | 3.10 (2.94–3.26) |
| 2009 | Census Region 1: Northeast | 2.42 (2.28–2.57) | 2009 | Census Region 2: Midwest | 3.07 (2.92–3.23) |
| 2010 | Census Region 1: Northeast | 3.00 (2.84–3.17) | 2010 | Census Region 2: Midwest | 3.05 (2.90–3.21) |
| 2011 | Census Region 1: Northeast | 3.09 (2.93–3.26) | 2011 | Census Region 2: Midwest | 3.22 (3.06–3.37) |
| 2012 | Census Region 1: Northeast | 3.21 (3.05–3.38) | 2012 | Census Region 2: Midwest | 3.25 (3.10–3.41) |
| 2013 | Census Region 1: Northeast | 3.21 (3.04–3.37) | 2013 | Census Region 2: Midwest | 3.35 (3.19–3.51) |
| 2014 | Census Region 1: Northeast | 3.15 (2.99–3.31) | 2014 | Census Region 2: Midwest | 3.40 (3.24–3.56) |
| 2015 | Census Region 1: Northeast | 3.19 (3.03–3.36) | 2015 | Census Region 2: Midwest | 3.33 (3.18–3.49) |
| 2016 | Census Region 1: Northeast | 2.95 (2.79–3.10) | 2016 | Census Region 2: Midwest | 3.45 (3.29–3.61) |
| 2017 | Census Region 1: Northeast | 3.27 (3.11–3.44) | 2017 | Census Region 2: Midwest | 3.79 (3.63–3.96) |
| 2018 | Census Region 1: Northeast | 3.38 (3.22–3.55) | 2018 | Census Region 2: Midwest | 3.80 (3.63–3.96) |
| 2019 | Census Region 1: Northeast | 3.37 (3.21–3.53) | 2019 | Census Region 2: Midwest | 4.02 (3.86–4.19) |
| 2020 | Census Region 1: Northeast | 4.06 (3.88–4.24) | 2020 | Census Region 2: Midwest | 4.84 (4.66–5.02) |
| 2021 | Census Region 1: Northeast | 4.65 (4.45–4.84) | 2021 | Census Region 2: Midwest | 5.27 (5.08–5.46) |
| 2022 | Census Region 1: Northeast | 4.93 (4.74–5.12) | 2022 | Census Region 2: Midwest | 5.16 (4.97–5.34) |
| 2023 | Census Region 1: Northeast | 4.72 (4.53–4.91) | 2023 | Census Region 2: Midwest | 5.29 (5.10–5.48) |
| **Year** | **Census Region** | **Age Adjusted Rate (95% CI)** | **Year** | **Census Region** | **Age Adjusted Rate (95% CI)** |
| 1999 | Census Region 3: South | 2.26 (2.14–2.38) | 1999 | Census Region 4: West | 1.83 (1.69–1.98) |
| 2000 | Census Region 3: South | 2.17 (2.05–2.29) | 2000 | Census Region 4: West | 2.02 (1.87–2.17) |
| 2001 | Census Region 3: South | 2.50 (2.38–2.62) | 2001 | Census Region 4: West | 2.29 (2.14–2.45) |
| 2002 | Census Region 3: South | 2.55 (2.42–2.67) | 2002 | Census Region 4: West | 2.39 (2.23–2.55) |
| 2003 | Census Region 3: South | 2.61 (2.48–2.74) | 2003 | Census Region 4: West | 2.53 (2.37–2.69) |
| 2004 | Census Region 3: South | 2.57 (2.44–2.69) | 2004 | Census Region 4: West | 2.49 (2.33–2.65) |
| 2005 | Census Region 3: South | 2.58 (2.45–2.70) | 2005 | Census Region 4: West | 2.46 (2.30–2.61) |
| 2006 | Census Region 3: South | 2.87 (2.74–3.00) | 2006 | Census Region 4: West | 2.52 (2.37–2.68) |
| 2007 | Census Region 3: South | 2.78 (2.66–2.91) | 2007 | Census Region 4: West | 2.67 (2.51–2.83) |
| 2008 | Census Region 3: South | 2.88 (2.76–3.01) | 2008 | Census Region 4: West | 2.67 (2.52–2.83) |
| 2009 | Census Region 3: South | 2.74 (2.62–2.87) | 2009 | Census Region 4: West | 2.65 (2.49–2.80) |
| 2010 | Census Region 3: South | 2.95 (2.83–3.07) | 2010 | Census Region 4: West | 2.82 (2.67–2.98) |
| 2011 | Census Region 3: South | 3.03 (2.90–3.15) | 2011 | Census Region 4: West | 2.95 (2.79–3.11) |
| 2012 | Census Region 3: South | 3.11 (2.99–3.23) | 2012 | Census Region 4: West | 3.04 (2.88–3.20) |
| 2013 | Census Region 3: South | 3.26 (3.14–3.39) | 2013 | Census Region 4: West | 2.95 (2.79–3.10) |
| 2014 | Census Region 3: South | 3.16 (3.04–3.28) | 2014 | Census Region 4: West | 3.05 (2.90–3.20) |
| 2015 | Census Region 3: South | 3.29 (3.17–3.41) | 2015 | Census Region 4: West | 2.97 (2.82–3.12) |
| 2016 | Census Region 3: South | 3.15 (3.03–3.27) | 2016 | Census Region 4: West | 3.06 (2.91–3.21) |
| 2017 | Census Region 3: South | 3.33 (3.21–3.45) | 2017 | Census Region 4: West | 3.22 (3.07–3.37) |
| 2018 | Census Region 3: South | 3.51 (3.39–3.63) | 2018 | Census Region 4: West | 3.31 (3.16–3.46) |
| 2019 | Census Region 3: South | 3.81 (3.69–3.93) | 2019 | Census Region 4: West | 3.52 (3.36–3.67) |
| 2020 | Census Region 3: South | 4.46 (4.33–4.59) | 2020 | Census Region 4: West | 4.03 (3.87–4.20) |
| 2021 | Census Region 3: South | 5.17 (5.03–5.32) | 2021 | Census Region 4: West | 4.77 (4.59–4.95) |
| 2022 | Census Region 3: South | 5.14 (5.00–5.28) | 2022 | Census Region 4: West | 4.83 (4.65–5.00) |
| 2023 | Census Region 3: South | 5.24 (5.10–5.38) | 2023 | Census Region 4: West | 4.69 (4.52–4.87) |

**Supplementary Table 5:** Urban and rural wise mortality data Involving Fatal-Arrhythmias and Anemia in the United States

| **Year** | **Area** | **Age adjusted rate (95% CI)** | **Year** | **Area** | **Age adjusted rate (95% CI)** |
| --- | --- | --- | --- | --- | --- |
| 1999 | Urban (Metropolitan) | 2.24 (2.17–2.32) | 1999 | Rural (Non-metropolitan) | 2.52 (2.35–2.69) |
| 2000 | Urban (Metropolitan) | 2.26 (2.19–2.34) | 2000 | Rural (Non-metropolitan) | 2.49 (2.33–2.66) |
| 2001 | Urban (Metropolitan) | 2.51 (2.43–2.59) | 2001 | Rural (Non-metropolitan) | 2.80 (2.63–2.98) |
| 2002 | Urban (Metropolitan) | 2.61 (2.52–2.69) | 2002 | Rural (Non-metropolitan) | 2.94 (2.76–3.12) |
| 2003 | Urban (Metropolitan) | 2.63 (2.55–2.72) | 2003 | Rural (Non-metropolitan) | 3.06 (2.88–3.25) |
| 2004 | Urban (Metropolitan) | 2.55 (2.47–2.63) | 2004 | Rural (Non-metropolitan) | 2.96 (2.78–3.14) |
| 2005 | Urban (Metropolitan) | 2.60 (2.52–2.68) | 2005 | Rural (Non-metropolitan) | 3.05 (2.87–3.24) |
| 2006 | Urban (Metropolitan) | 2.69 (2.61–2.77) | 2006 | Rural (Non-metropolitan) | 3.18 (3.00–3.37) |
| 2007 | Urban (Metropolitan) | 2.69 (2.61–2.77) | 2007 | Rural (Non-metropolitan) | 3.21 (3.03–3.39) |
| 2008 | Urban (Metropolitan) | 2.74 (2.66–2.82) | 2008 | Rural (Non-metropolitan) | 3.30 (3.12–3.49) |
| 2009 | Urban (Metropolitan) | 2.61 (2.53–2.69) | 2009 | Rural (Non-metropolitan) | 3.36 (3.18–3.55) |
| 2010 | Urban (Metropolitan) | 2.86 (2.78–2.94) | 2010 | Rural (Non-metropolitan) | 3.41 (3.23–3.60) |
| 2011 | Urban (Metropolitan) | 2.96 (2.88–3.04) | 2011 | Rural (Non-metropolitan) | 3.67 (3.47–3.86) |
| 2012 | Urban (Metropolitan) | 3.06 (2.98–3.14) | 2012 | Rural (Non-metropolitan) | 3.66 (3.47–3.85) |
| 2013 | Urban (Metropolitan) | 3.15 (3.07–3.23) | 2013 | Rural (Non-metropolitan) | 3.52 (3.34–3.71) |
| 2014 | Urban (Metropolitan) | 3.06 (2.98–3.14) | 2014 | Rural (Non-metropolitan) | 3.75 (3.56–3.94) |
| 2015 | Urban (Metropolitan) | 3.07 (3.00–3.15) | 2015 | Rural (Non-metropolitan) | 3.91 (3.72–4.11) |
| 2016 | Urban (Metropolitan) | 3.05 (2.98–3.13) | 2016 | Rural (Non-metropolitan) | 3.70 (3.51–3.88) |
| 2017 | Urban (Metropolitan) | 3.26 (3.18–3.33) | 2017 | Rural (Non-metropolitan) | 4.08 (3.89–4.28) |
| 2018 | Urban (Metropolitan) | 3.35 (3.28–3.43) | 2018 | Rural (Non-metropolitan) | 4.31 (4.11–4.50) |
| 2019 | Urban (Metropolitan) | 3.56 (3.48–3.64) | 2019 | Rural (Non-metropolitan) | 4.52 (4.32–4.72) |
| 2020 | Urban (Metropolitan) | 4.14 (4.05–4.22) | 2020 | Rural (Non-metropolitan) | 5.63 (5.41–5.85) |

**Supplementary Table 6:** State specified mortality data Involving Fatal-Arrhythmias and Anemia in the United States

| **State** | **Age adjusted rate (95% CI)** | **State** | **Age adjusted rate (95% CI)** |
| --- | --- | --- | --- |
| Alabama | 2.66 (2.54–2.78) | Montana | 2.81 (2.56–3.06) |
| Alaska | 2.75 (2.29–3.21) | Nebraska | 3.76 (3.54–3.97) |
| Arizona | 1.47 (1.39–1.54) | Nevada | 1.28 (1.16–1.41) |
| Arkansas | 2.79 (2.64–2.94) | New Hampshire | 3.15 (2.90–3.39) |
| California | 3.26 (3.21–3.31) | New Jersey | 4.08 (3.98–4.18) |
| Colorado | 2.48 (2.36–2.61) | New Mexico | 1.67 (1.52–1.81) |
| Connecticut | 2.35 (2.23–2.47) | New York | 1.89 (1.84–1.94) |
| Delaware | 3.35 (3.05–3.65) | North Carolina | 3.45 (3.35–3.55) |
| District of Columbia | 2.68 (2.33–3.03) | North Dakota | 4.08 (3.73–4.43) |
| Florida | 1.98 (1.93–2.02) | Ohio | 4.80 (4.70–4.90) |
| Georgia | 1.65 (1.58–1.73) | Oklahoma | 3.16 (3.02–3.31) |
| Hawaii | 2.88 (2.66–3.10) | Oregon | 3.20 (3.06–3.34) |
| Idaho | 2.34 (2.13–2.54) | Pennsylvania | 3.86 (3.78–3.94) |
| Illinois | 2.53 (2.46–2.60) | Rhode Island | 5.11 (4.80–5.42) |
| Indiana | 3.72 (3.60–3.84) | South Carolina | 4.28 (4.12–4.43) |
| Iowa | 3.17 (3.02–3.32) | South Dakota | 3.11 (2.83–3.39) |
| Kansas | 2.51 (2.36–2.65) | Tennessee | 3.52 (3.40–3.64) |
| Kentucky | 3.87 (3.72–4.02) | Texas | 4.37 (4.29–4.44) |
| Louisiana | 1.77 (1.66–1.87) | Utah | 1.46 (1.32–1.60) |
| Maine | 2.89 (2.68–3.10) | Vermont | 4.61 (4.20–5.01) |
| Maryland | 4.39 (4.25–4.53) | Virginia | 2.45 (2.36–2.55) |
| Massachusetts | 2.14 (2.06–2.23) | Washington | 3.77 (3.65–3.90) |
| Michigan | 2.54 (2.46–2.62) | West Virginia | 4.82 (4.58–5.05) |
| Minnesota | 4.28 (4.14–4.42) | Wisconsin | 2.74 (2.63–2.84) |
| Mississippi | 2.27 (2.13–2.42) | Wyoming | 3.07 (2.68–3.45) |
| Missouri | 2.78 (2.68–2.89) |  |  |
